# Artificial Intelligence Can Cut Costs While Maintaining Accuracy in Colorectal Cancer Genotyping

**DOI:** 10.3389/fonc.2021.630953

**Published:** 2021-06-08

**Authors:** Alec J. Kacew, Garth W. Strohbehn, Loren Saulsberry, Neda Laiteerapong, Nicole A. Cipriani, Jakob N. Kather, Alexander T. Pearson

**Affiliations:** ^1^ Pritzker School of Medicine, University of Chicago, Chicago, IL, United States; ^2^ Department of Medicine, University of Chicago, Chicago, IL, United States; ^3^ Department of Public Health Sciences, University of Chicago, Chicago, IL, United States; ^4^ Department of Pathology, University of Chicago, Chicago, IL, United States; ^5^ Department of Medicine, University Hospital Rheinisch-Westfälische Technische Hochschule (RWTH) Aachen, Aachen, Germany

**Keywords:** deep learning, microsatellite instability (MSI), colorectal (colon) cancer, financial implication, digital biomarker, digital pathology, cost savings, artificial intelligence

## Abstract

Rising cancer care costs impose financial burdens on health systems. Applying artificial intelligence to diagnostic algorithms may reduce testing costs and avoid wasteful therapy-related expenditures. To evaluate the financial and clinical impact of incorporating artificial intelligence-based determination of mismatch repair/microsatellite instability status into the first-line metastatic colorectal carcinoma setting, we developed a deterministic model to compare eight testing strategies: A) next-generation sequencing alone, B) high-sensitivity polymerase chain reaction or immunohistochemistry panel alone, C) high-specificity panel alone, D) high-specificity artificial intelligence alone, E) high-sensitivity artificial intelligence followed by next generation sequencing, F) high-specificity artificial intelligence followed by next-generation sequencing, G) high-sensitivity artificial intelligence and high-sensitivity panel, and H) high-sensitivity artificial intelligence and high-specificity panel. We used a hypothetical, nationally representative, population-based sample of individuals receiving first-line treatment for *de novo* metastatic colorectal cancer (N = 32,549) in the United States. Model inputs were derived from secondary research (peer-reviewed literature and Medicare data). We estimated the population-level diagnostic costs and clinical implications for each testing strategy. The testing strategy that resulted in the greatest project cost savings (including testing and first-line drug cost) compared to next-generation sequencing alone in newly-diagnosed metastatic colorectal cancer was using high-sensitivity artificial intelligence followed by confirmatory high-specificity polymerase chain reaction or immunohistochemistry panel for patients testing negative by artificial intelligence ($400 million, 12.9%). The high-specificity artificial intelligence-only strategy resulted in the most favorable clinical impact, with 97% diagnostic accuracy in guiding genotype-directed treatment and average time to treatment initiation of less than one day. Artificial intelligence has the potential to reduce both time to treatment initiation and costs in the metastatic colorectal cancer setting without meaningfully sacrificing diagnostic accuracy. We expect the artificial intelligence value proposition to improve in coming years, with increasing diagnostic accuracy and decreasing costs of processing power. To extract maximal value from the technology, health systems should evaluate integrating diagnostic histopathologic artificial intelligence into institutional protocols, perhaps in place of other genotyping methodologies.

## Introduction

Oncologic diagnostic algorithms, specifically those involving next-generation sequencing (NGS), financially burden healthcare systems. Just as the advent of NGS was an advancement over polymerase chain reaction (PCR) and immunohistochemistry (IHC) for some applications, artificial intelligence (AI) may be the next innovative oncologic diagnostic agent. From routine histopathology images, AI can recapitulate genetic information with area under the receiver-operator curve (ROC) approaching 0.9 (1, 2). AI may help overcome NGS-related challenges like cost, packing and shipping delays, and turnaround time. Due to massive scalability, AI costs, following initial investment, would be a fraction of other technologies’ costs. Since tumors grow in the absence of treatment, AI’s faster turnaround (and associated earlier treatment initiation) could impact clinical outcomes.

AI may be especially impactful in common malignancies. In the United States (U.S.), nearly 150,000 cases of colorectal cancer (CRC) are diagnosed annually (3). In the metastatic setting (22% of cases), deficient mismatch repair (dMMR) or high microsatellite instability (MSI-H) – genetic features seen in 5% of metastatic CRC (mCRC) cases – are predictive and prognostic (4). For individuals with dMMR/MSI-H mCRC, KEYNOTE-177 demonstrated superior outcomes for front-line pembrolizumab over cytotoxic chemotherapy (5). The high price of immunotherapies (like pembrolizumab) could portend a significant escalation in the total cost of mCRC care. Diagnostic strategies can limit immunotherapy use to only those patients who are most likely to benefit. Like NGS, PCR, and IHC, AI, although not currently part of routine clinical practice, can infer actionable genetic features like MMR/MSI, *KRAS*, and *BRAF* status from histopathology (1, 2). In the present study, we projected the financial and clinical impacts of incorporating AI into the diagnostic algorithm. We are unaware of any prior research in estimating the financial impact of implementing AI in a clinical context – this study is the first one in our knowledge to do so. Our results, along with future real-world confirmation across cancers, could inform policy and practice to optimize oncologic diagnostic pathways.

## Materials and Methods

**Figure 1:**
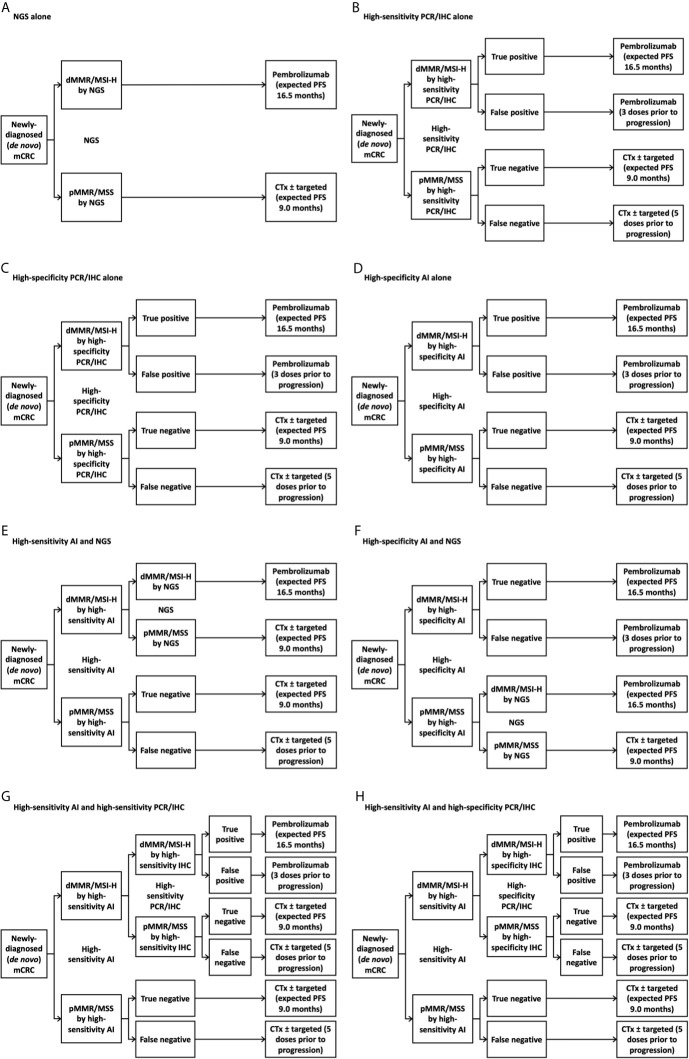
Treatment decision tree with various testing strategies. **(A)** Next-generation sequencing alone; **(B)** High-sensitivity immunohistochemistry panel alone; **(C)** High-specificity immunohistochemistry panel alone; **(D)** High-specificity artificial intelligence alone; **(E)** High-sensitivity artificial intelligence followed by next-generation sequencing for individuals with deficient mismatch repair/microsatellite instability-high tumors by artificial intelligence; **(F)** High-specificity artificial intelligence followed by next-generation sequencing for individuals with intact mismatch repair/microsatellite stable tumors by artificial intelligence; **(G)** High-sensitivity artificial intelligence followed by high-sensitivity immunohistochemistry panel for individuals with deficient mismatch repair/microsatellite instability-high tumors by artificial intelligence; **(H)** High-sensitivity artificial intelligence followed by high-specificity immunohistochemistry panel for individuals with deficient mismatch repair/microsatellite instability-high tumors by artificial intelligence. AI, artificial intelligence; CTx, chemotherapy; dMMR, deficient mismatch repair; FU, fluorouracil; IHC, immunohistochemistry; mCRC, metastatic colorectal cancer; MSI-H, microsatellite instability-high; MSS, microsatellite-stable; NGS, next-generation sequencing; PCR, polymerase chain reaction; PFS, progression-free survival; pMMR, proficient mismatch repair.

We generated eight potential diagnostic algorithms for determining MMR/MSI status in the U.S. first-line newly-diagnosed (*de novo*) mCRC population: NGS alone, high-sensitivity PCR or IHC panel (“panel” for short) alone, high-specificity panel alone, high-specificity AI alone, high-sensitivity AI with confirmatory NGS for patients testing negative by AI, high-specificity AI with confirmatory NGS for patients testing positive by AI, high-sensitivity AI with confirmatory high-sensitivity panel for patients testing positive by AI, and high-sensitivity AI with confirmatory high-specificity panel for patients testing positive by AI (**Figure 1**). We chose these diagnostic scenarios based on current standard-of-care and, based on clinical and cost considerations, where AI might reasonably fit within the diagnostic paradigm. We took “NGS alone” as the reference approach, as it was expected to be the costliest, and chose other scenarios to include clinically reasonable permutations of using NGS, panel, and/or AI. We evaluated costs from the perspective of the U.S. healthcare system, agnostic of payer. We assessed costs over one year, as longer time horizons might not account for yet-unknown future changes in oncologic technology and practice over longer timeframes. Given the relatively short time horizon, our model did not incorporate a discount rate. Consideration of opportunity costs (e.g., potential use of cost-savings derived from new diagnostic approaches) was outside our scope.

**Figure 2:**
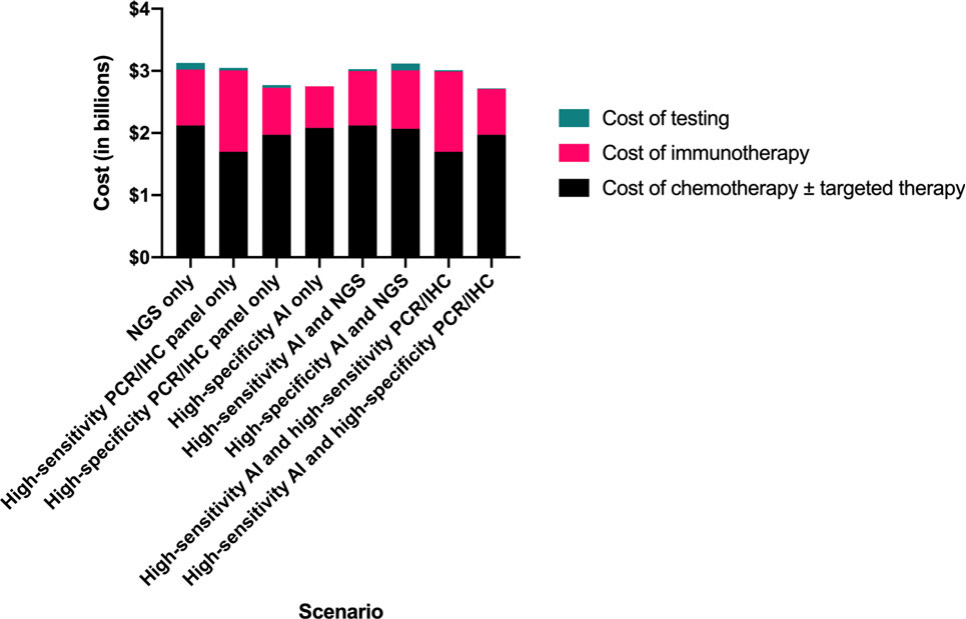
Comparison of total testing and treatment-related costs by clinical scenario. AI, artificial intelligence; IHC, immunohistochemistry; NGS, next-generation sequencing; PCR, polymerase chain reaction.

We incorporated data from peer-reviewed literature and government sources into a financial and clinical model (**Table 1** and **Figure 2**) (6, 7, 10–14). All cost assumptions were based on values reported in 2017-2020. We aimed to use as few data sources as possible in the interest of minimizing heterogeneity of assumptions. We gathered nearly all dollar values from publicly available reimbursement schedules of the Centers for Medicare & Medicaid Services. Test characteristics of the AI platform is based on our group’s previous work. Absolute population and incidence estimates were derived from the Surveillance, Epidemiology, and End Results Program (SEER) database, while proportions of patients falling into genetic and treatment subgroups was derived from a variety of peer-reviewed publications. The timing of restaging scans was based on the restaging cadence in the KEYNOTE-177 trial. We chose the AI sensitivity/specificity cutoffs based on two points along our previously-developed ROC (**Figure 1**) (2). Briefly, we developed our AI algorithm using hematoxylin and eosin-stained slides for samples that had previously been analyzed for MSI-H/dMMR status by either IHC or PCR. Pathologists who had been blinded to clinical data and MSI-H/dMMR status determined sample quality and area of tumor tissue. Images were saved digitally, color-normalized, then subjected to our deep learning system. We did not incorporate the cost of developing the deep learning model into our financial estimate, as we have already developed this approach.

**Table 1:** Model assumptions and inputs.

Model input	Assumed value (reference)
**Population characteristics**	
# newly diagnosed colorectal cancer per year in the U.S.	147,950 (3)
% metastatic	22% (3)
# newly diagnosed (*de novo*) metastatic colorectal cancer per year in the U.S.	32,549
% dMMR/MSI-H	5% (4)
% pMMR/MSS	95% (4)
**Diagnostic characteristics**	
Cost per patient of next-generation sequencing	$3,500.00 (6)
Cost per patient of PCR or IHC panel	$1,206.25
KRAS/NRAS	$682.29 (6)
BRAF	$175.40 (6)
dMMR/MSI-H	$348.56 (6)
Cost per patient of artificial intelligence (digital image scanning)	$6.07^a^
Time for next-generation sequencing (days)	12 (7)
Time for PCR or IHC panel (days)	4 (7)
Time for artificial intelligence (months) – assumed nominal value	-
Next generation sequencing sensitivity – conservative assumption	100%
Next generation sequencing specificity – conservative assumption	100%
PCR or IHC dMMR/MSI-H panel sensitivity (high sensitivity cutoff):	100% (8)
PCR or IHC dMMR/MSI-H panel specificity (high sensitivity cutoff):	81% (8)
PCR or IHC dMMR/MSI-H panel sensitivity (high specificity cutoff):	67% (9)
PCR or IHC dMMR/MSI-H panel specificity (high specificity cutoff):	93% (9)
Artificial intelligence dMMR/MSI-H sensitivity (high sensitivity cutoff)	98% (2)
Artificial intelligence dMMR/MSI-H specificity (high sensitivity cutoff)	79% (2)
Artificial intelligence dMMR/MSI-H sensitivity (high specificity cutoff)	70% (2)
Artificial intelligence dMMR/MSI-H specificity (high specificity cutoff)	98% (2)
**Therapeutic characteristics**	
Cost per patient per month for dMMR/MSI-H therapy	$23,021.13 (5)
Weighted average cost per patient per month of 5-fluorouracil-based therapy^b^	$7,625.88
% receiving FOLFOX + bevacizumab	35% (10)
Cost per patient per month for FOLFOX + bevacizumab	$6,316.70 (11)
% receiving FOLFOX + cetuximab	45% (10)
Cost per patient per month for FOLFOX + cetuximab	$11,945.73 (11)
% receiving 5-fluorouracil + leucovorin	20% (10)
Cost per patient per month for 5-fluorouracil + leucovorin	$179.76 (11)
Weighted average cost per patient per dose of 5-fluorouracil-based therapy	$3,807.68
% receiving FOLFOX + bevacizumab	35% (10)
Cost per patient per dose for FOLFOX + bevacizumab	$3,158.35 (11)
% receiving FOLFOX + cetuximab	45% (10)
Cost per patient per dose for FOLFOX + cetuximab	$5,972.86 (11)
% receiving 5-fluorouracil + leucovorin	20% (12)
Cost per patient per dose for 5-fluorouracil + leucovorin	$63.63 (11)
Weighted average median time on of 5-fluorouracil-based therapy (months)	9.0
% receiving FOLFOX + bevacizumab	35% (10)
Median time on therapy for FOLFOX + bevacizumab	10.3 (13)
% receiving FOLFOX + cetuximab	45% (10)
Median time on therapy for FOLFOX + cetuximab	10 (13)
% receiving 5-fluorouracil + leucovorin	20% (12)
Median time on therapy for 5-fluorouracil + leucovorin	4.4 (14)
Time between scans (months)	2.07 (5)
Number of pembrolizumab doses before first restaging scans	3
Time between pembrolizumab doses (months)	0.69 (5)
Number of chemotherapy ± targeted therapy doses before first restaging scans	5
Time between chemotherapy ± targeted therapy doses (months)	0.46 (5)

Superscript numbers represent references. Values without references are calculated from other values in the table unless otherwise noted.

a Internal, documentation available upon request.

b Among patients with pMMR/MSS disease, we assumed that patients ineligible for intensive therapy would receive 5-fluorouracil (5-FU) and leucovorin (LV). Among the remaining patients with pMMR/MSS disease, we assumed that all patients with RAS wild type disease would receive 5-FU, LV, oxaliplatin (FOLFOX), and cetuximab, while all patients with RAS mutant disease would receive FOLFOX and bevacizumab.

dMMR, deficient mismatch repair; FOLFOX, 5-fluorouracil, leucovorin, oxaliplatin; IHC, immunohistochemistry; MSI-H, microsatellite instability-high; MSS, microsatellite-stable; PCR, polymerase chain reaction; pMMR, proficient mismatch repair; U.S., United States.

We grouped PCR and IHC and assessed two sets of test characteristics (high-specificity and high-sensitivity) for these platforms, as characteristics vary across studies (8, 9). Our primary objective was to compare total costs of testing and first-line therapy across the scenarios. To assess clinical impact of each diagnostic strategy, we estimated time to treatment initiation, proportion of patients receiving results within guideline-recommended 10 working days from laboratory sample receipt (15), and proportion of patients receiving first-line therapy supported by KEYNOTE-177. Since we did not have a direct way of linking these clinical consequences with clinical outcomes, we did not compare incremental cost-effectiveness ratios.

## Results

We projected the high-sensitivity AI followed by high-specificity panel strategy to result in the lowest total testing and first-line drug therapy cost, $2.72 billion, compared to $3.13 billion for NGS alone, representing savings of $400 million (12.9%) (**Table 2**). The high-specificity panel-only and the high-specificity AI-only scenarios resulted in nearly as much cost savings ($360 million and $370 million, respectively).

**Table 2:** Cost of therapy and clinical impact by diagnostic strategy.

	NGS only	High-sensitivity PCR/IHC only	High-specificity PCR/IHC only	High-specificity AI only	High-sensitivity AI and NGS	High-specificity AI and NGS	High-sensitivity AI and high-sensitivity PCR/IHC	High-sensitivity AI and high-specificity PCR/IHC
Total cost of diagnostic testing and first-line therapy	$3.13	$3.05	$2.76	$2.75	$3.03	$3.12	$3.00	$2.72
Cost of chemotherapy ± targeted therapy	$2.12	$1.70	$1.97	$2.08	$2.12	$2.07	$1.70	$1.97
Cost of immunotherapy	$0.90	$1.31	$0.76	$0.67	$0.88	$0.94	$1.29	$0.74
Cost of testing	$0.11	$0.04	$0.04	$0.00	$0.03	$0.11	$0.02	$0.01
Cost savings compared to reference scenario (NGS only) (absolute)	*Reference*	$0.07	$0.36	$0.37	$0.10	$0.01	$0.12	$0.40
Cost savings compared to reference scenario (NGS only) (relative)	*Reference*	2.3%	11.6%	11.9%	3.2%	0.2%	3.9%	12.9%
Weighted average time to treatment initiation	12	4	4	0	3.0	11.4	1.6	1.2
Percent of patients receiving results within guideline-recommended 10 working days (15)	0%	100%	100%	100%	75%	5%	100%	100%
Percent of patients receiving first-line therapy supported by KEYNOTE-177	100%	81%	91%	97%	80%	97%	81%	91%

Dollar values presented in billions. AI, artificial intelligence; IHC, immunohistochemistry; NGS, next-generation sequencing; PCR, polymerase chain reaction.

The high-specificity AI-only scenario was associated with the shortest time to treatment initiation (<1 day) (versus 12 days for NGS), with 100% of patients receiving results within the guideline-recommended ten working days (versus 0% for NGS). Compared with the NGS-only scenario, in which all 32,549 (100%) patients received KEYNOTE-177-supported therapy, 31,442 of 32,549 (97%) patients received KEYNOTE-177-supported therapy in the high-specificity AI-only scenario.

We estimate that the accuracy of AI is similar to the accuracies of PCR and IHC in determining MSI/MMR status. For the high-sensitivity context (i.e., as screening tests), we estimate 98% sensitivity and 79% specificity for AI compared to 100% sensitivity and 81% specificity for PCR/IHC. In the high-specificity context (i.e., as confirmatory tests), we estimate 70% sensitivity and 98% specificity for AI compared to 67% sensitivity and 93% specificity for PCR/IHC.

## Discussion

The $400 million (12.9%) difference between the most and least expensive scenarios highlights that testing approach can significantly impact costs in the setting of first line mCRC. The least costly scenario, high-sensitivity AI with confirmatory high-specificity panel, comes with the tradeoff of 9% of patients (2,815) receiving a first-line therapy not supported by KEYNOTE-177 data (versus 0% with NGS-only). The second-least costly scenario, using high-specificity AI alone, results in only 3% of patients (1,107) receiving a non-supported therapy. It is our view that the ability to start therapy earlier due to elimination of treatment initiation delay (e.g., time for packing and shipping of tissue samples to outside facilities, time to conduct tests) may compensate, to some degree, for any reduction in median progression-free survival (PFS) resulting from that 3%. Moreover, the Kaplan-Meier PFS curves from KEYNOTE-177 suggest that PFS for pembrolizumab and chemotherapies are similar for the first eight months of therapy. Only after this timepoint do the curves separate, disease tends to progress (PFS 8.2 months), and patients will likely switch therapy. We could draw the conclusion, then, that chemotherapy offers similar benefit to pembrolizumab in dMMR/MSI-H disease for several months. If we accept this premise, at least in part, then perhaps treating 1,107 patients with a non-KEYNOTE-177-supported therapy, and avoiding additional immunotherapy cost, becomes more reasonable. If we consider where else in the health system the $400 million in savings could be spent, the prospect becomes more palatable still.

It is important to acknowledge that established tests are only as powerful as the biomarkers that they assess. While 43.8% of dMMR/MSI-H patients respond to pembrolizumab, health systems would benefit from diagnostic tools that could help avoid using costly immunotherapy in the dMMR/MSI-H patients who will not respond (i.e., the majority of these patients). With potential to consider tumor characteristics beyond genetics (e.g., intratumoral heterogeneity, three-dimensional structure), AI could prove to be even more predictive than NGS. In other words, the promise of AI is not to be a cost-effective approximating of existing technologies, but rather an improvement upon them, both in terms of clinical utility and cost.

It is important to recognize that applying artificial intelligence to digital histopathology is only one cog in a much broader wheel of strategies to curb healthcare spending. For example, screening for early detection of colorectal cancer is another vital component of a greater program to curb costs, as screening is estimated to be associated with $1.50 to $2.00 in returns for each dollar spent (16). Uptake of cost-cutting measures like AI relies on appropriate financial incentives presented to hospitals and clinics. Whereas classic buy-and-bill outpatient reimbursement actually encourages overspending (as the reimbursement is pegged to the cost of the purchase), structures like the oncology care model encourage providers to make choices that curb costs. Health systems must seek to target multiple levers (e.g., at the levels of screening, diagnosis, and treatment) to achieve financial sustainability in oncologic health. Besides, any dollar saved from one sector within the field of oncologic health can be routed towards spending on those areas with the highest value (e.g., investment in screening).

The main limitation of our study was the use of a theoretical model, which will require real-world validation. The integrity of our estimates is dependent on the validity of the sources that we used to develop input assumptions. Although we aimed to use as few sources as possible to allow for some standardization among or assumptions, our assumptions are derived from a diverse range of sources. We aimed, too, to use data from high-quality, prospective clinical trials, where possible, but there were numerous cases in which the required data was only available in the form of retrospective analyses. Since the application of AI to histopathology diagnostics has not been widely used in clinical contexts, we do not yet have access to real-world cost and outcomes data. Our model did not consider important aspects like heterogeneity in the population and varying costs by setting and payer, instead assuming a monolithic U.S. healthcare system for demonstrative purposes. Each individual institution’s initial fixed costs associated with implementing digital histopathology are also outside of the scope of our study. These costs might include purchasing or renting hardware (e.g., slide scanners) and software (e.g., cloud data storage) from digital histopathology vendors. On an ongoing basis, additional pathology personnel would likely be required to perform new tasks like internal validation, maintaining hardware and software, and scanning slides. However, multiple previous analyses have suggested that gains in efficiency and productivity associated with implementing digital histopathology more than pay for these upfront and ongoing costs (17, 18). There may be additional costs of which we are not currently aware, as potential costs may arise in the real world that have not been encountered before, given the novelty of this platform. It is important to be conscientious, too, of more abstract implementation hurdles like earning clinicians’ confidence in new technologies. NGS, IHC, and PCR are trusted tools on which clinicians have long relied for guiding treatment decisions. Encouraging the adoption of a technology unlike any of the current diagnostic tools may be an uphill battle in some contexts. Finally, any new tool of this kind must undergo rigorous validation to ensure that in offers equal benefit across demographic groups (e.g., by race, ethnicity, socio-economic status). Our study did not account for any such heterogeneity. Our conclusions would benefit greatly from validation with future independent cohorts.

While we used first-line therapy for mCRC as an example, we view these findings as relevant across cancers whose diagnostic algorithm involves genetic evaluation – with savings far beyond this sliver of total spending on cancer care. Not only would the initial investment in AI eventually pay for itself, but, because of the nature of the technology, AI improves as the platform “learns” from each sample. In this way, every dollar spent on AI is an investment in a better technology. This valuable characteristic of AI differentiates it and positions it as a vehicle for improving the quality and cost of cancer care.

## Data Availability Statement

The original contributions presented in the study are included in the article/**Supplementary Material**. Further inquiries can be directed to the corresponding authors.

## Author Contributions

AK - Performed analysis and wrote manuscript. GS, LS, NL, and NC - Provided analytical advice and edited manuscript. JK - Formulated research question, designed analysis, supervised research, and edited manuscript. AP - Formulated research question, designed analysis, supervised research, edited manuscript, and provided resources and funding. All authors contributed to the article and approved the submitted version.

## Funding

JK is funded by the Max-Eder-Programme of the German Cancer Aid (Bonn, Germany, grant # 70113864) and the START Programme of the Medical Faculty Aachen (Aachen, Germany, grant #691906). AP is funded by NIH/NIDCR K08-DE026500 and NIH/NCI U01-CA243075, and has research funding from the Adenoid Cystic Carcinoma Research Foundation, Cancer Research Foundation, and University of Chicago Comprehensive Cancer Center. NL is funded by NIMHD R01MD013420 and NIDDK P30 DK092949.

## Conflict of Interest

The authors declare that the research was conducted in the absence of any commercial or financial relationships that could be construed as a potential conflict of interest.
