# Supplementary figures and images for: Artificial Intelligence Can Cut Costs While Maintaining Accuracy in Colorectal Cancer Genotyping
Source: Front Oncol. 2021 Jun 8;11:630953. doi: 10.3389/fonc.2021.630953 (PMC8217761; doi:10.3389/fonc.2021.630953)

**eFigure 1: Artificial intelligence receiver-operator curve and choice of cutoffs**

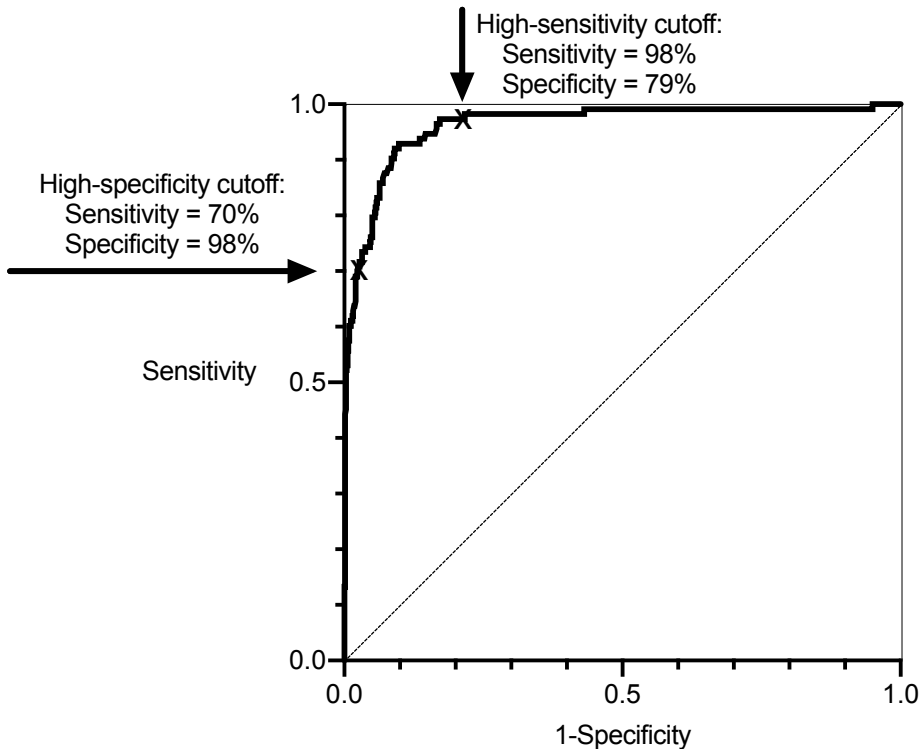

Supplement: Supplementary file 1 [file DataSheet_1.pdf]
